# Impact of the second reader on screening outcome at blinded double reading of digital screening mammograms

**DOI:** 10.1038/s41416-018-0195-6

**Published:** 2018-07-24

**Authors:** Angela M. P. Coolen, Adri C. Voogd, Luc J. Strobbe, Marieke W. J. Louwman, Vivianne C. G. Tjan-Heijnen, Lucien E. M. Duijm

**Affiliations:** 1 Department of Radiology, Elisabeth-Tweesteden Hospital (ETZ), PO Box 90151, 5000 LC Tilburg, The Netherlands; 2 Department of Epidemiology, GROW, Maastricht University, P Debyelaan 1, 6229 HA Maastricht, The Netherlands; 3 Department of Research, Netherlands Comprehensive Cancer Organization (IKNL), PO Box 19079, 3501 DB Utrecht, The Netherlands; 4 Department of Surgery, Canisius-Wilhelmina Hospital, PO Box 9015, 6500 GS Nijmegen, The Netherlands; 5 Department of Internal Medicine, Division of Medical Oncology, GROW, Maastricht University Medical Centre, P Debyelaan 25, 6229 HX Maastricht, The Netherlands; 6 Department of Radiology, Canisius Wilhelmina Hospital, Weg door Jonkerbos 100, 6532 SZ Nijmegen, The Netherlands; 7 Dutch Expert Centre for Screening, Wijchenseweg 101, 6538 SW Nijmegen, The Netherlands

**Keywords:** Population screening, Breast cancer, Epidemiology

## Abstract

**Background:**

To determine the impact of the second reader on screening outcome at blinded double reading of digital screening mammograms.

**Methods:**

We included a consecutive series of 99,013 digital screening mammograms, obtained between July 2013 and January 2015 and double read in a blinded fashion. During 2-year follow-up, we collected radiology, surgery and pathology reports of recalled women.

**Results:**

Single reading resulted in 2928 recalls and 616 screen-detected cancers (SDCs). The second reader recalled another 612 women, resulting in 82 additional SDCs. Addition of the second reader increased the recall rate (3.0% to 3.6%, *p* < 0.001), cancer detection rate (6.2–7.0 per 1000 screens, *p* < 0.001) and false positive recall rate (24.4–28.7 per 1000 screens, *p* < 0.001). Positive predictive value of recall (21.0% vs. 19.7%, *p* = 0.20) and of biopsy (52.1% vs. 50.9%, *p* = 0.56) were comparable for single reading and blinded double reading. Tumour characteristics were comparable for cancers detected by the first reader and cancers additionally detected by the second reader, except of a more favourable tumour grade in the latter group.

**Conclusions:**

At blinded double reading, the second reader significantly increases the cancer detection rate, at the expense of an increased recall rate and false positive recall rate.

## Introduction

The first regional and nationwide breast cancer screening programmes were implemented in the 1980’s.^1^ These screening programmes aimed to reduce breast cancer mortality through detection and treatment of breast malignancies at an early stage. In the Netherlands, the nationwide biennial breast cancer screening programme was gradually implemented from 1989 through 1997.^2^ Years later screen-film mammography (SFM) was replaced by full-field digital mammography (FFDM), significantly enhancing the cancer detection rate (CDR).^3,4^ In our screening region, the transition to FFDM was completed in 2010. Twenty years after the introduction of biennial mammography screening in the Netherlands, breast cancer mortality has declined by 30–34%.^5^ The reported breast cancer mortality before the implementation of the Dutch nationwide breast cancer screening programme was 91.6%. This was reduced to 75.4% in the SFM period and further declined to 55.1% in 2014.^6^ This mortality reduction is attributed to the combination of an earlier detection of breast cancer and significant improvements in breast cancer treatment.^5^

Reading strategies used to assess screening mammograms are single reading, with or without computer aided detection (CAD), and double reading. Double reading can be performed in either a non-blinded or blinded fashion. At non-blinded (or ‘independent’) double reading, the second reader is aware of the first reader’s opinion, whereas the second reader is blinded to the opinion of the first reader in case of blinded double reading. In conjunction with European guidelines,^7^ double reading is standard of care in the Dutch nationwide breast cancer screening programme. Studies dating from the era of SFM have shown that non-blinded double reading significantly increases the CDR compared to single reading.^8,9^ With the introduction of digital mammography, blinded double reading became technically feasible. Klompenhouwer et al. compared blinded and non-blinded double reading and found a higher CDR and programme sensitivity at blinded double reading, at the expense of an increased recall rate and false positive recall rate.^10^ In the current study we prospectively determined screening outcome at blinded double reading vs. single reading in a biennial digital screening mammography programme in a southern region of the Netherlands.

## Materials and Methods

### Study population

In this prospective study we used information from 99,013 consecutive screening examinations (9860 initial screens and 89,143 subsequent screens, respectively) performed between 1 July 2013 and 1 January 2015. These FFDM were obtained at four specialised screening units in a southern biennial screening mammography region of the Netherlands (BOZ, Bevolkings Onderzoek Zuid). The Dutch breast cancer screening programme targets women aged 50–75. On entering the Dutch nationwide screening programme, all women are routinely asked to give permission to use their data for scientific purposes. One woman refused this permission and was therefore excluded from our study. This study was performed within the national permit for breast cancer screening and did not require an additional permit, according to the Dutch Law on Population-based screening.

### Screening procedure and recall

Details of the Dutch nationwide breast cancer screening programme have been described previously.^11^ In brief, mammograms were acquired by certified mammographers using a Lorad Selenia FFDM system (Hologic Inc, Danbury, CT), with a 70 µm pixel size and a 232 × 286 mm field of view.

All 99,013 mammographic examinations were double read in a blinded fashion by a team of 13 certified screening radiologists, each of them reading at least 10,000 mammograms per year. Thus, when the first reader decided to recall, the mammogram was always read by a second screening radiologist. Mammograms were classified according to the Breast Imaging Reporting and Data System (BI-RADS).^12^ Previously obtained mammograms were available for comparison in case of a subsequent screening. Although in the Dutch screening programme, after performing the mammographic examination, the mammographer annotates whether she would recall a women, mammographers do not function as official readers. The radiologists were not blinded to the mammographers opinion.

Mammographic abnormalities were classified as either a suspicious mass, suspicious microcalcifications, suspicious mass with microcalcifications, architectural distortion, asymmetry or other abnormality. A discordant reading was defined as a difference in classification by two readers, where one reader classified the mammogram BI-RADS 1 or 2 (negative, i.e. no recall) and the other reader classified it as either BI-RADS 0, 4 or 5 (positive, i.e. recall). All other cases were classified as concordant readings. In addition to all concordant BI-RADS 0, 4 and 5 mammograms, all discordant readings were recalled (i.e. no arbitration took place for discordant readings).

For training purposes, every six weeks, a supervising breast radiologist discussed all recall decisions made by the screening radiologists, with the mammographers. In addition, all cases with a negative reading at radiologist blinded double reading but a positive mammographer reading were re-assessed by the supervising breast radiologist. A woman was recalled at this stage if the supervising radiologist considered workup necessary.

### Diagnostic workup and follow-up after recall

In case of a positive screening result, the woman was referred by her general practitioner to the breast unit of one of the nearby hospitals. The general practitioner and the hospital breast unit were only informed about the type of mammographic abnormality and corresponding BI-RADS classification and were blinded to the type of recall (discordant vs. concordant). After physical examination by a surgical oncologist or dedicated breast nurse, additional mammographic and/or tomosynthesis views were obtained at the clinical radiologist’s discretion. The previous screening mammograms were routinely available and stored in the Picture Archiving and Communication System of the hospital. All mammograms were classified according to BI-RADS. Dependent on the outcome of both the physical examination and mammography, further workup consisted of one or a combination of the following: breast ultrasonography (US), MR imaging and/or biopsy. During a follow-up period of about 2 years (until the next biennial screening examination) screening mammography findings, clinical data, additional clinical imaging reports, biopsy reports and surgery reports were collected of all recalled women. Breast cancers were divided in ductal carcinoma in-situ (DCIS) and invasive cancers. Lobular carcinoma in-situ (LCIS) was considered to be a benign lesion.

### Statistical analysis

The primary outcome measures of our study were recall rate (recalls per 100 screens), CDR (CDR, screen-detected cancers per 1000 screens), interval cancer rate (ICR, interval cancers per 1000 screens), false positive rate (FPR, false positive recalls per 1000 screens), positive predictive value (PPV) of recall and PPV of biopsy. These outcome measures were compared for single reading and blinded double reading using McNemar’s test (recall rate, CDR and FPR) and Chi-square test (PPV of recall and biopsy). A Chi-square test was performed to test differences in type of mammographic abnormality, BI-RADS classification at recall, diagnosis after recall (true positive vs. false positive), type of screen-detected cancer (DCIS vs. invasive cancer) and also tumour stage and other tumour characteristics (size, histopathologic type and grading, hormone receptor status, lymph node status) of tumours detected by single reading and blinded double reading. In case of bilateral disease the tumour with the most advanced tumour stage was included in the analysis. When multiple foci of cancer were found in the same breast, only the largest tumour was taken into account for analysis. The significance level was set at 5%. Statistical analysis was performed using IBM SPSS Statistics 23.0 (IBM SPSS Statistics for Windows, Version 23.0. Armonk, NY: IBM Corp).

## Results

### Overall screening results

**Fig. 1:**
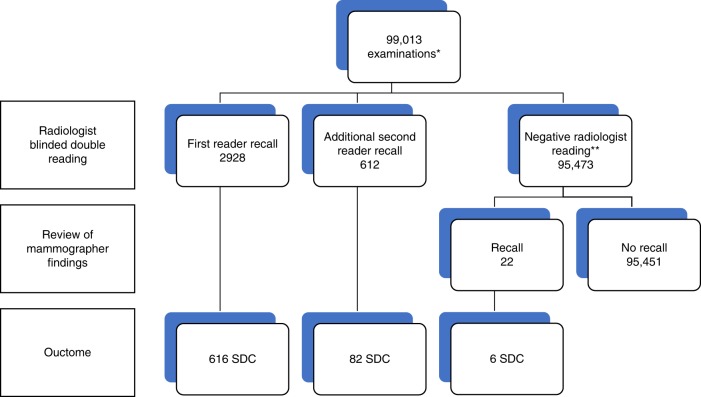
Radiologist single reading vs. radiologist double reading: mammography screening outcome at 2-year follow-up. *All 99,013 mammographic examinations were read by two certified screening radiologists in a blinded fashion. First reader recalls comprise all positive readings by the first reader (concordant and discordant recalls). Additional second reader recalls are positive readings by the second reader (by definition discordant recalls, i.e. negative reading by the first reader). ** In case of a negative reading by both radiologists and a positive mammographer reading, a women was recalled if the supervising radiologist considered workup obligatory. These 22 women were excluded from statistical analysis. SDC screen-detected cancer

Out of 99,013 screened women, 3562 were recalled for further evaluation of a mammographic abnormality (recall rate, 3.6%). Breast cancer was diagnosed in 704 women, resulting in an overall CDR of 7.1 per 1000 screens and a PPV of recall of 19.8% (Fig. 1). The false positive recall rate was 28.9 per 1000 screens (2858/99,013). We traced a total of 162 interval cancers, resulting in an interval cancer rate of 1.6 per 1000 screens. Programme sensitivity was 81% (704/866). Twenty-two women were recalled after re-assessment of positive mammographer findings, resulting in six additional screen-detected cancers. Since the current study focusses on radiologist blinded double reading, these 22 recalls and 6 additional cancers were hereafter excluded from statistical analysis.

### Single reading vs. blinded double reading

The first radiologist recalled a total of 2928/99,013 women (recall rate 3.0%). Another 612/99,013 women were recalled by the second radiologist, resulting in a significantly higher recall rate at blinded double reading compared to single reading (3.6% vs. 3.0%, *p* < 0.001, Table 1). The CDR also significantly increased from 6.2 (616/99,013) per 1000 screens at single reading to 7.0 (698/99,013) at blinded double reading (*p* < 0.001), with a 13.3% relative increase in CDR. The false positive rate per 1000 screens increased from 23.4 (2312/99,013) at single reading to 28.7 (2842/99,013) at blinded double reading (*p* < 0.001). The positive predictive value of recall (21.0% (616/2928) vs. 19.7% (698/3540), *p* = 0.20) and positive predictive value of biopsy (52.1% (616/1128) vs. 50.9% (698/1372), *p* = 0.56) were comparable for single reading and blinded double reading.

**Table 1:** Screening outcome at single reading vs. blinded double reading

	Single reading	Blinded double reading	*P*-value
Recalls, no (recall rate)	2928 (3.0)	3540 (3.6)	<0.001
Screen-detected cancers, no (CDR)	616 (6.2)	698 (7.0)	<0.001
False positives, no (FPR)	2312 (23.4)	2842 (28.7)	<0.001
Positive predictive value of recall, %	21.0	19.7	0.20
Positive predictive value of biopsy, %	52.1	50.9	0.56

*CDR* cancer detection rate (number of cancers detected per 1000 screens), *FPR* false positive rate (number of false positive recalls per 1,000 screens)

The proportion of BI-RADS 0 lesions was significantly higher in women additionally recalled by the second reader (74.5% vs. 62.2%, *p* < 0.001), in combination with significantly fewer BI-RADS 4 and 5 recalls (25.5% vs. 37.8%, *p* < 0.001) (Table 2). The lesions additionally recalled by the second reader comprised larger proportions of asymmetries and architectural distortions than masses and/or microcalcifications when compared to the proportions of various mammographic abnormalities recalled by the first reader (*p* < 0.001, Table 2). Moreover, for lesions additionally recalled by the second reader, the type of assessment more frequently consisted of breast imaging only, without biopsy (*p* = 0.001, Table 2). Finally, these lesions comprised more false positive recalls and fewer cancers compared to those lesions recalled by the first reader (*p* < 0.001, Table 2).

**Table 2:** Mammographic characteristics and outcome of lesions recalled by the 1^st^ reader vs. lesions additionally recalled by the 2^nd^ reader

	Recalls by 1^st^ reader (*n* = 2928)	Additional recalls by 2^nd^ reader (*n* = 612)	*P*-value
Mammographic abnormality^a^			
Mass	2084 (71.2)	427 (69.8)	<0.001
Microcalcifications	429 (14.7)	75 (12.3)	
Mass with microcalcifications	106 (3.6)	14 (2.3)	
Asymmetry	119 (4.1)	29 (4.7)	
Architectural distortion	190 (6.5)	67 (10.9)	
BI-RADS at recall			
BI-RADS 0	1820 (62.2)	285 (75.4)	<0.001
BI-RADS 4 or 5	1108 (37.8)	93 (24.6)	
Type of assessment after recall			
None	10 (0.3)	0 (0)	0.001
Clinical breast imaging	1736 (59.3)	422 (69.0)	
Clinical breast imaging + biopsy	1182 (40.4)	190 (31.0)	
Screening outcome			
False positive	2312 (79.0)	530 (86.6)	<0.001
True positive	616 (21.0)	82 (13.4)	

^a^Dominant mammographic abnormality in case of multiple recalled lesions

### Tumour characteristics of screen-detected cancers

The tumour characteristics of cancers detected by the first reader were comparable to those of cancers additionally detected by the second reader, except of the tumour grade of invasive cancers (Table 3). The proportion of well differentiated, invasive cancers (Bloom & Richardson grade I) was larger in the group of 61 invasive cancers additionally detected by the second reader (59.0% vs. 39.8%, *p* = 0.021, Table 3). The final surgical treatment (breast conserving surgery vs. mastectomy) also did not differ significantly between both groups (Table 3).

**Table 3:** Tumour characteristics of cancers detected by the 1st reader and of cancers additionally detected by the 2nd reader at blinded double reading

	Cancers detected by 1^st^ reader (*n* = 616)	Cancers additionally detected by 2^nd^ reader (*n* = 82)	*P*-value
Mammographic abnormality			
Mass	388 (63.0)	39 (47.6)	0.113
Microcalcifications	129 (20.9)	28 (34.1)	
Mass with microcalcifications	52 (8.4)	6 (7.3)	
Asymmetry	7 (1.1)	1 (1.2)	
Architectural distortion	40 (6.5)	8 (9.8)	
Type of cancer			
DCIS	108 (17.5)	21 (25.6)	0.110
Invasive	508 (82.5)	61 (74.4)	
DCIS grade			
Low	17 (15.7)	5 (23.8)	0.616
Intermediate	39 (36.1)	9 (42.9)	
High	52 (48.1)	7 (33.3)	
Histology of invasive cancers			
Ductal	407 (80.1)	47 (77.0)	0.368
Lobular	54 (10.6)	4 (6.6)	
Mixed ductal/lobular	15 (3.0)	4 (6.6)	
Other	32 (6.3)	6 (9.8)	
Tumour size of invasive cancers			
T1 (≤20 mm)	404 (79.5)	53 (86.9)	0.103
T2+(>20 mm)	104 (20.5)	8 (13.1)	
Lymph node status of invasive cancers			
N+	117 (23.0)	11 (18.0)	0.324
N-	383 (75.4)	48 (78.7)	
Unknown	8 (1.6)	2 (3.3)	
Bloom & Richardson grade			
I	202 (39.8)	36 (59.0)	0.021
II	236 (46.5)	16 (26.2)	
III	63 (12.4)	9 (14.8)	
Unknown	7 (1.4)	0	
Oestrogen receptor status			
Positive	460 (90.6	56 (91.8)	0.819
Negative	45 (8.9)	5 (8.2)	
Unknown	3 (0.6)	0	
Progesterone receptor status			
Positive	369 (72.6)	39 (63.9)	0.378
Negative	136 (26.8)	22 (36.1)	
Unknown	6 (1.2)	0	
Her2/Neu receptor status			
Positive	44 (8.7)	8 (13.1)	0.292
Negative	458 (90.2)	53 (86.9)	
Unknown	6 (1.2)	0	
Final surgical treatment			
Breast conserving surgery	494 (80.2)	61 (74.4)	0.534
Mastectomy	114 (18.5)	20 (24.4)	
No surgery	8 (1.3)	1 (1.2	

*DCIS* ductal carcinoma in-situ

## Discussion

The current study reports on the impact of the second reader on screening outcome at blinded double reading of screening mammograms in a southern region of the Dutch nationwide breast cancer screening programme. Compared to single reading, addition of a second reader significantly increased the CDR, at the expense of a significantly increased recall rate and false positive recall rate.

Breast screening programmes aim to reduce patient morbidity and mortality through the detection of early-stage breast cancers, with acceptable false positive recall rates. This balance between CDR and false positive recall rate is a very delicate one.^10^ Compared to single reading, addition of the second reader significantly increased the CDR from 6.2 to 7.0 per 1000 screens, at the expense of a significant increase in recall rate, from 3.0% to 3.6%. The higher recall rate at blinded double reading, observed in our study, is in line with studies reported by Posso et al.^13,14^ However, these authors found a comparable CDR of 4.2–4.8 and 4.6–5.2 per 1000 screens at single reading and double reading. The significant increase in CDR that we observed after second reading may be largely explained by a difference in screening programme design but also by the fact that we included more screens. Posso et al. included women aged 50–69 years and performed consensus with arbitration of discordant readings. Moreover, their study was characterised by higher recall rates (4.6–4.8%), much lower CDRs and a PPV of recall of less than 10%. Our recall rate of 3.6% is still within the ranges of the European guidelines for quality assurance^7^ and the Dutch optimisation study of Otten et al.^15^

A majority of the 82 cancers additionally detected by the second reader (11.7% of all cancers detected by the screening radiologists) were small (T1a-c) invasive cancers (64.6%) or DCIS (25.6%), and most invasive cancers were of low histological grade. As blinded double reading detects additional cancers at an early stage, one may assume that this reading strategy will further reduce breast cancer morbidity and mortality. However, recent studies suggest that some of these small invasive cancers with favourable biological behaviour do not progress to clinically significant cancers during the lifetime of the patient and therefore actually represent overdiagnosis.^16,17^

There were significantly more BI-RADS 0 recalls among women additionally recalled by the second reader than among women recalled after single reading, with a larger proportion of women who underwent imaging only after recall by the second reader. In the Dutch screening setting, BI-RADS 0 represents a mammographic finding needing additional workup and it is generally considered to be a lesion with a relatively low malignancy risk, of ~7%.^18^ In the Netherlands, nearly 70% of women with a BI-RADS 0 recall undergo non-invasive assessment only to confirm the benign nature of their abnormality detected at screening mammography, without the need of any additional biopsy procedures.^19^ A possible explanation for the differences in recall BI-RADS would be that the percentage of BI-RADS 0 is higher in case of a discordant reading because two readers are more likely to agree on a more obvious (BI-RADS 4 or 5) abnormality. With our study design, additional recalls at blinded double reading are by definition discordant readings. The second reader might however not agree with all readings by the first reader (i.e. there are also discordant readings in the group women recalled by the first reader). Differences in screening outcome and tumour characteristics between discordant vs. concordant recalls at blinded double reading are a subject of further study.

Unlike Posso et al. we did not perform a cost-effectiveness analysis. In 2014, the total cost per screening examination was €66^6^ and only a small proportion (less than 10%) of these costs were spent on the screening radiologists. In the Dutch breast cancer screening programme it is therefore not likely that a change of reading strategy from double to single reading would result in a significant cost reduction. Since Posso et al. did not find a significant increase in CDR with double reading, it is not surprising that they concluded that double reading is not a cost-effective strategy.

Our study has several strengths and limitations. It is a large study, with virtually complete follow-up, that focuses on the impact of a second reader on screening outcome. On the other hand, as mentioned earlier, our report does not provide information on the cost-effectiveness of blinded double reading, a topic that is of importance for further study in order to make a definite recommendation. Second, extrapolation of our results to other screening programmes may be limited by the fact that the design of the Dutch breast cancer screening programme (blinded double reading) and workup strategies differ from other countries. Third, our study design does not allow comparison of interval cancer rates between single and blinded double reading. Another limitation of our study design is that we cannot be sure screening radiologists would read in the same way when placed in a situation where there is no safety net of a second reader. Finally, we did not investigate the influence of arbitration of discordant readings on screening outcome.

In conclusion, we favour blinded double reading over single reading as the first reading strategy significantly increases the CDR, at the expense of an acceptable increase in recall rate and false positive recall rate. Further research on cost-effectiveness is needed to make a more definite recommendation.

## **Availability of data and materials**

The data that support the findings of this study are available from the corresponding author upon reasonable request.
